# A real-world study of palbociclib plus endocrine therapy with or without a short course chemotherapy in the first-line treatment of HR-positive HER2-negative metastatic breast cancer

**DOI:** 10.3389/fonc.2025.1512496

**Published:** 2025-07-09

**Authors:** Xiangjun Li, Yuhua Song, Meng Lv, Yongmei Wang, Xueqiang Gao, Tianyi Ma, Teng Ma, Changgen Liu, Xinyi Sun, Haibo Wang, Yan Mao

**Affiliations:** Breast Disease Center, Affiliated Hospital of Qingdao University, Qingdao, Shandong, China

**Keywords:** metastatic breast cancer, palbociclib, endocrine treatment, cyclin-dependent kinases 4/6 inhibitor, chemotherapy

## Abstract

**Background:**

To investigate the efficacy of palbociclib plus endocrine therapy (ET) as the initial treatment compared with post-chemotherapy maintenance therapy in the first-line treatment of hormone receptor-positive (HR-positive), human epidermal growth factor receptor 2-negative (HER2-negative) metastatic breast cancer (MBC).

**Methods:**

A total of 110 patients with HR-positive HER2-negative MBC were enrolled in this study between 2018 and 2023. Progression-free-survivals (PFS) and Overall Survival (OS) of palbociclib plus ET as the initial treatment (group A, n:78) or as post-chemotherapy maintenance therapy (group B, n:32) were calculated. We used the multivariable Cox model to investigate the relationship between each factor and prognosis and performed subgroup analysis.

**Results:**

The median duration of follow-up across the cohort was 45.3 months (95% CI, 42.7 to 50.9 months) in all patients. Statistical analysis revealed no significant difference in PFS between the two groups (*p*=0.21). 50% was the objective response rate (ORR) for both groups. The disease control rate (DCR) for group A was 95.1% (95%CI 0.88 to 0.98), and for group B, it was 100% (95% CI 0.89 to 1.00). Multivariate Cox regression analysis indicated that the initial administration of palbociclib plus ET was significantly correlated with improved OS (Hazard Ratio [HR] = 0.36, 95% CI, 1.20 to 11.14, *p* < 0.05).

**Conclusion:**

This real-world study revealed that the commencement of therapy with palbociclib in synergy with ET was preferable to effective chemotherapy followed by palbociclib plus ET.

## 1 Introduction

In 2024, there will likely be 611,720 cancer deaths and 2,001,140 new cancer cases in the United States (1), despite a discernible decline in the aggregate cancer mortality rate. Among all female cancers, breast cancer has the greatest incidence rate, which has a substantial financial impact on families and society (2). Within the spectrum of breast cancer phenotypes, the predominant subtype is hormone receptor-positive (HR-positive) cancer, comprising approximately 70% of all breast cancer cases (3, 4). For perimenopausal or premenopausal women exhibiting HR-positive, human epidermal growth factor receptor 2 (HER2)-negative metastatic breast cancer (MBC), the standard initial therapy comprises endocrine therapy (ET), augmented by ovarian suppression or ablation.

While ET confers benefits to the majority of patients with advanced HR-positive breast cancer, approximately 20% may exhibit primary resistance. Furthermore, an additional 30% to 40% of patients are at risk of disease progression due to secondary resistance (5). The advent of cyclin-dependent kinases 4/6 (CDK4/6) inhibitors (6–8) has significantly altered the landscape of ET, with the continuous advancement of molecular biology and tumor ecology research. A large number of biomarkers (9–12) and therapeutic targets have been gradually uncovered, leading to more in-depth research into the precision and stratified treatment of breast cancer. Consequently, ET combined with CDK4/6 inhibitor has emerged as the preferred treatment option for patients with metastatic HR-positive breast cancer (13, 14).

Palbociclib (15–18), a pioneering CDK4/6 inhibitor, has garnered approval for clinical use within China, marking a significant milestone in treating breast cancer. Contemporary clinical trials have substantiated that for patients with advanced breast cancer devoid of visceral crisis, ET is the primary therapeutic recommendation. The efficacy of combining CDK4/6 inhibitors with ET has been demonstrated to surpass that of ET in isolation. Nevertheless, for patients requiring prompt intervention to arrest the advancement of non-visceral crises, chemotherapy is deemed a feasible alternative, owing to the delayed therapeutic onset characteristic of endocrine therapy (19, 20). Presently, evidence is lacking to conclusively determine whether using CDK4/6 inhibitors plus ET as the initial treatment or after stabilization with chemotherapy (21), is optimal for certain cohorts of HR-positive, HER2-negative MBC patients.

Hence, this study proposed to conduct real-world research, for HR-positive, HER2-negative MBC patients who have not received any rescue treatment in the past. Subjects will be stratified based on their actual therapy into two groups: one receiving palbociclib plus ET as the initial treatment, and another as maintenance therapy after effective chemotherapy. The objective is to compare the efficacy of the two groups’ treatments, analyze the characteristics of the dominant population benefiting from each group’s treatment, and provide a basis for the first-line treatment choice for HR-positive, and HER2-negative MBC patients.

## 2 Methods

### 2.1 Patients and study criteria

The medical records of patients with HR-positive, HER2-negative metastasis breast cancer at the Breast Disease Center in the Affiliated Hospital of Qingdao University between August 2018 and August 2023 were retrospectively collected. The inclusion criteria were as follows (1): Pathological testing of the primary lesion in female patients with invasive breast cancer shows HR-positive, HER2 negative. Since there is proof of metastasis, they cannot be cured by radiation therapy or surgical excision. (a) ER-positive and/or PR positive is defined as the proportion of tumor cells with positive staining accounting for ≥1% of all tumor cells (22) (to be verified by the pathology department of the research center involved in this study). (b) HER2-negative is defined as a standard immunohistochemical (IHC) test of 0/1+, ISH detection the HER2/CEP17 ratio is less than 2.0 or the HER2 gene copy number is less than 4. Based on this, HER2-low is typically classified as IHC 1+ or IHC 2+ with negative ISH; HER2-zero corresponds to IHC 0 (2). Have not received any prior systemic anticancer therapy for advanced disease (3). Possess quantifiable lesions or only metastatic lesions limited to bone that satisfy Response Evaluation Criteria in Solid Tumors 1.1 (RECIST 1.1) criteria, such as mixed or osteolytic lesions (4). The first-line treatment plan involves the selection of palbociclib in combination with initial endocrine therapy or as maintenance therapy following chemotherapy. The exclusion criteria were as follows (1): Patients received prior treatment with chemotherapy for advanced disease, fulvestrant, or a CDK4/6 inhibitor (2). Previous endocrine therapy was used in the advanced setting (3). Considerable loss or lack of medical records during follow-up.

Patients were classified according to metastatic pattern as follows: Synchronous disease: Refers to the presence of metastases that are detected at the same time as, or within 3 months of, the diagnosis of the primary tumor. Metachronous disease: Refers to metastases that are detected more than 3 months after the diagnosis of the primary tumor, indicating disease progression over time. Local treatments (surgery and radiotherapy) were applied exclusively to the primary tumor. For patients with metachronous disease, these treatments were administered at the time of initial diagnosis, prior to the detection of distant metastases. In contrast, for patients with synchronous disease, local treatments were delivered after the diagnosis of metastatic disease and were thus considered palliative in intent.

Patients were classified according to endocrine therapy response (23) as follows: Primary endocrine resistance was defined as relapse during the first 2 years of adjuvant endocrine therapy, or disease progression within the first 6 months of first-line endocrine-based treatment for advanced breast cancer. Secondary endocrine resistance referred to relapse after the first 2 years of adjuvant endocrine therapy, or within 12 months after completing adjuvant therapy, or disease progression after at least 6 months of endocrine treatment in the metastatic setting. Endocrine sensitivity was defined as the absence of any prior exposure to endocrine therapy, or disease recurrence occurring at least 12 months after completion of adjuvant endocrine therapy.

A total of 356 patients were diagnosed with HR-positive, HER2-negative metastasis breast cancer during this period, of which 110 satisfied the inclusion-exclusion criteria. This study was approved by the Ethics Committee of the Qingdao University Affiliated Hospital of (QYFYKYLL 969311920). Before participation, each patient provided written informed consent and was followed up until March 15, 2024.

### 2.2 Data collection

**Figure 1:**
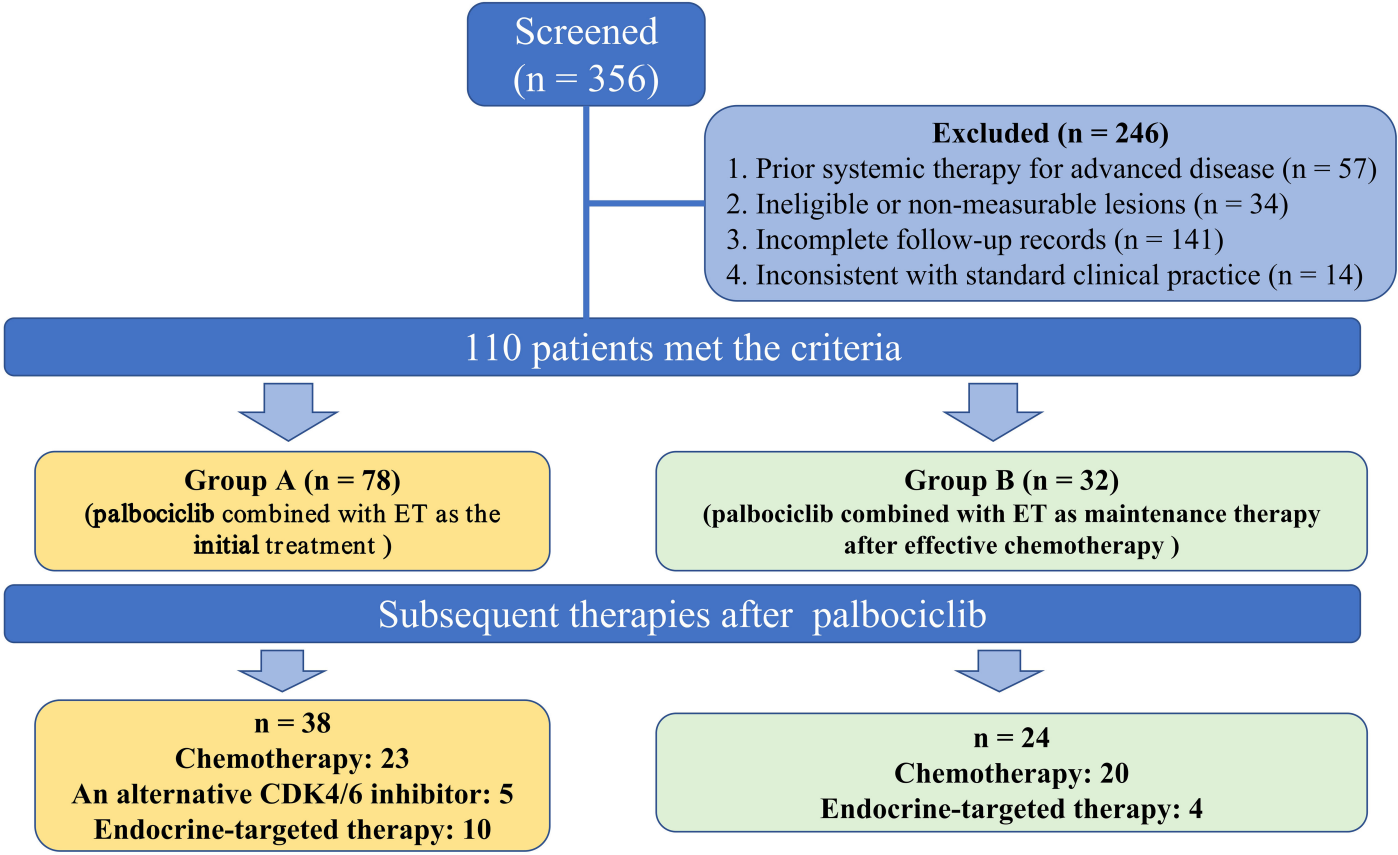
The research flowchart.

Each patient’s clinicopathological characteristics and prognostic count data were obtained from medical records and during follow-up visits. The clinical characteristics included age, menstrual status, Eastern Cooperative Oncology Group Performance Status (ECOG PS), pathologic staging, ER status, PR status, HER2 status, Tumor-node-metastasis (TNM) stage, Ki67 expression, date of diagnosis metastasis, combined endocrine therapy, median duration of palbociclib, treatments they received after palbociclib, and dates of progression under treatment. A total of 110 patients included in the study were evaluated in two groups (**Figure 1**): those who received palbociclib plus ET as the initial treatment (group A, n: 78) and those who using palbociclib with post-chemotherapy maintenance therapy (group B, n: 32). The median Progression-Free Survival (PFS) of group A and group B were compared. In addition, the median PFS of different second-line treatment regimens after palbociclib progression were compared.

### 2.3 Statistical analyses

Chi-square tests were used to analyze whether the baseline distributions of the two groups of patients were balanced. The Kaplan-Meier method was used to estimate survival rates by group and to plot survival curves. The stratified Log-Rank method was used to compare the survival functions of the two groups. The Kaplan-Meier method was used to estimate the median PFS, and OS (Overall Survival). In this study, secondary evaluation indicators were calculated: disease control rate (DCR) and objective response rate (ORR). PFS is defined as the time from the date of enrolment to the date of first documented objective disease progression (according to RECIST 1.1) or the date of any cause-related death, whichever happened first. DCR is the sum of the complete response (CR), partial response (PR), and stable disease (SD). ORR, the proportion of subjects with CR and PR from the date of advanced first-line treatment to the duration of treatment, according to RECIST 1.1. The Cox proportional hazards model was used to estimate the hazard ratio between groups and its 95% confidence interval. We used multiple Cox regression models to investigate the relationship between each factor and prognosis and performed subgroup analysis according to age, menopausal status, and metastases. Subgroup analysis was carried out to identify more beneficiaries.

## 3 Results

### 3.1 Baseline characteristics of patients treated with palbociclib plus ET

The initial demographic and clinical characteristics demonstrated equitable distribution across between two groups as delineated in **Table 1**, with 78 in Group A and 32 in Group B. The mean age was comparable between the two groups (55.95 vs. 55.62 years, *p* = 0.898). No significant difference was observed in menopausal status, pathological subtype, HER2 expression level, Ki-67 index (*p* > 0.05). Additionally, the number of metastatic lesions was more limited in Group A, with 55.1% presenting with a single metastatic site compared to only 18.8% in Group B (*p* = 0.002). Visceral metastases were significantly more frequent in Group B (75.0% vs. 43.6%, *p* = 0.005), while bone-only metastases tended to be more prevalent in Group A (*p* = 0.052). Regarding treatment history, 65.4% of patients in Group A and 56.2% in Group B received radiotherapy (*p* = 0.416), while prior chemotherapy was reported in 71.6% and 81.2% of patients, respectively (*p* = 0.356). In terms of ET response, 29.6% of patients in Group A were classified as ET-sensitive, 51.9% exhibited secondary resistance, and 18.5% had primary resistance. A similar distribution was observed in Group B, with 31.2% ET-sensitive, 56.2% secondarily resistant, and 12.5% primarily resistant patients (*p* = 0.855). The choice of endocrine agents combined with palbociclib included fulvestrant (33.6%), letrozole (23.9%), exemestane (22.1%), and anastrozole (20.4%), with no significant difference in distribution between the two groups (*p* = 0.237).

**Table 1:** Baseline characteristics.

Variable	Total (N = 110)	Group A (n = 78)	Group B (n = 32)	P-value
Age, mean (SD)	55.85 (12.39)	55.95 (12.12)	55.62 (13.22)	0.898
Menopausal status				0.619
Postmenopausal	63 (57.3%)	43 (55.1%)	20 (62.5%)	
Premenopausal	47 (42.7%)	35 (44.9%)	12 (37.5%)	
ECOG				<0.001
0	59 (53.6%)	51 (65.4%)	8 (25.0%)	
1	44 (40.0%)	24 (30.8%)	20 (62.5%)	
2	7 (6.4%)	3 (3.8%)	4 (12.5%)	
Metastatic pattern				0.273
Metachronous disease	91 (82.7%)	67 (85.9%)	24 (75.0%)	
Synchronous disease	19 (17.3%)	11 (14.2%)	8 (25.0%)	
Pathological type				0.134
IDC	106 (96.3%)	74 (94.9%)	32 (100.0%)	
ILC	4 (3.6%)	4 (5.1%)	0 (0.0%)	
PR				0.045
Positive	90 (81.8%)	68 (87.2%)	22 (68.8%)	
Negative	20 (18.2%)	10 (12.8%)	10 (31.2%)	
HER2				0.240
Zero	31 (28.2%)	25 (32.1%)	6 (18.8%)	
Low	79 (71.8%)	53 (67.9%)	26 (81.2%)	
Ki67				0.247
≤ 30%	79 (71.8%)	59 (75.6%)	20 (62.5%)	
> 30%	32 (28.2%)	19 (24.4%)	12 (37.5%)	
Metastasis lesions				0.002
1	49 (44.5%)	43 (55.1%)	6 (18.8%)	
2	25 (22.7%)	15 (19.2%)	10 (31.2%)	
≥ 3	36 (32.7%)	20 (25.6%)	16 (50.0%)	
Bone metastasis				0.052
No	58 (52.7%)	36 (46.2%)	22 (68.8%)	
Yes	52 (47.3%)	42 (53.8%)	10 (31.2%)	
Visceral metastasis				0.005
No	52 (47.3%)	44 (56.4%)	8 (25.0%)	
Yes	58 (52.7%)	34 (43.6%)	24 (75.0%)	
Surgery on primary tumor				0.090
Yes	94 (85.5%)	70 (89.7%)	24 (75.0%)	
No	16 (14.5%)	8 (10.3%)	8 (25.0%)	
Radiotherapy				0.416
Yes	71 (62.8%)	53 (65.4%)	18 (56.2%)	
No	42 (37.2%)	28 (34.6%)	14 (43.8%)	
Chemotherapy				0.356
Yes	84 (74.3%)	58 (71.6%)	26 (81.2%)	
No	29 (25.7%)	23 (28.4%)	6 (18.8%)	
Sensitivity to endocrine therapy				0.855
Primary resistance	19 (16.8%)	15 (18.5%)	4 (12.5%)	
Secondary resistance	60 (53.1%)	42 (51.9%)	18 (56.2%)	
Sensitive	34 (30.1%)	24 (29.6%)	10 (31.2%)	
Combined endocrine therapy				0.237
Anastrozole	23 (20.4%)	17 (21.0%)	6 (18.8%)	
Exemestane	25 (22.1%)	14 (17.3%)	11 (34.4%)	
Fulvestrant	38 (33.6%)	28 (34.6%)	10 (31.2%)	
Letrozole	27 (23.9%)	22 (27.2%)	5 (15.6%)	

bbreviations: ECOG, Eastern Cooperative Oncology Group performance status; ER, estrogen receptor; PR, progesterone receptor; HER2, human epidermal growth factor receptor 2.

### 3.2 Survival outcomes

**Figure 2:**
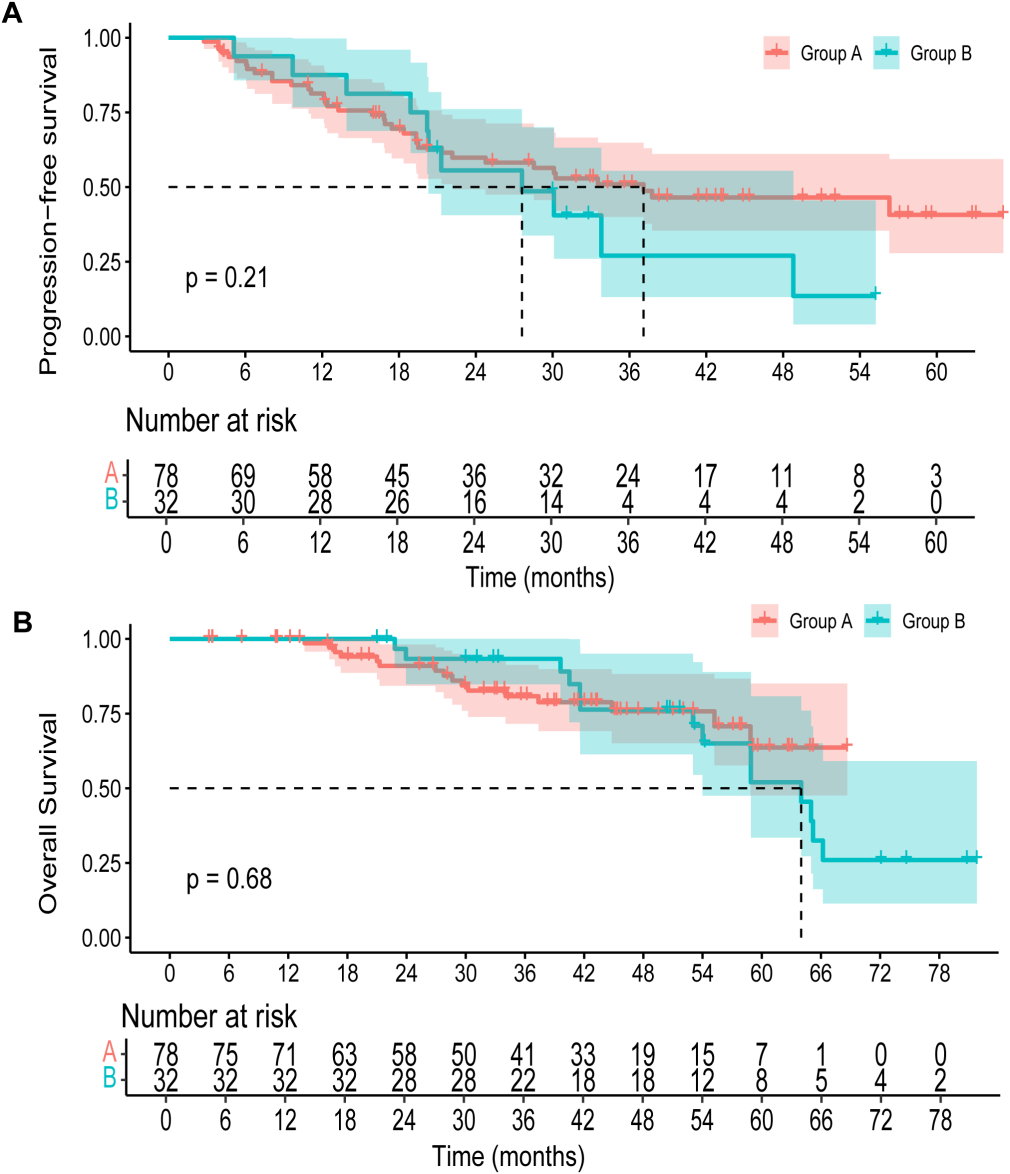
Kaplan–Meier survival curve of **(A)** progression-free survival, **(B)** overall survival.

The median duration of follow-up was 45.3 months (95% CI, 42.7 to 50.9 months) for all patients. The median PFS was 37.1 months (95% CI, 22.2 to NA months) in group A versus 27.6 months (95% CI, 20.3 to 48.8 months) in group B. No significant difference in PFS was observed between the two groups (*p*=0.21) (**Figure 2A**). The 3-year OS rate was 80.8% (95% CI, 71.6 to 91.3) in group A and 93.3% (95% CI, 84.8 to 100.0) in group B. By 5 years, the OS rate declined to 63.7% (95% CI, 47.6 to 85.1) in group A and 52.0% (95% CI, 33.5 to 80.8) in group B (**Figure 2B**). Upon data cutoff, with 16 fatalities in group A and 14 in group B, OS showed no discernible difference (*p*=0.68). The objective response rate (ORR) was comparable between the two groups, with 50.0% in group A (95% CI, 0.39 to 0.61) and 50.0% in group B (95% CI, 0.30 to 0.68) (**Table 2**). The disease control rate (DCR) was 94.9% in group A (95% CI, 0.88 to 0.98), while all patients in group B achieved disease control (DCR = 100.0%, 95% CI, 0.89 to 1.00).

**Table 2:** Best overall response in all patients.

Best overall response	Group A (N=78)	Group B (N=32)
CR	9 (11.5%)	2 (6.3%)
PD	4 (5.1%)	0 (0.0%)
PR	30 (38.5%)	14 (43.8%)
SD	35 (44.9%)	16 (50.0%)
ORR	39 (50.0%)	16 (50.0%)
95% CI	0.39 to 0.61	0.30 to 0.68
DCR	74 (94.9%)	32 (100.0%)
95% CI	0.88 to 0.98	0.89 to 1.00

CR, complete response; PD, Progressive Disease;

PR, partial response; SD, stable disease; ORR, objective response rate;

DCR, disease control rate.

In the univariate analysis (**Table 3**), ECOG performance status, PR expression, number of metastatic lesions, visceral metastasis, radiotherapy history and treatment group were significantly associated with overall survival. Specifically, patients with ECOG 2 exhibited a higher risk of death compared to those with ECOG 0 (HR = 5.23, 95% CI: 1.47-18.67, p = 0.011). PR positivity was associated with improved survival (HR = 0.42, 95% CI: 0.20-0.88, p = 0.022), while patients with ≥3 metastatic lesions had poorer outcomes (HR = 3.10, 95% CI: 1.21-7.93, p = 0.018). Visceral metastasis (HR = 5.02, p = 0.001) and history of radiotherapy (HR = 3.14, p = 0.010) were also correlated with inferior survival. In the multivariate model, ECOG 2 remained an independent predictor of worse survival (HR = 4.99, 95% CI: 1.17-21.29, *p* = 0.024). Visceral metastasis also independently predicted poorer prognosis (HR = 4.85, 95% CI: 1.52-15.42, *p* = 0.013). Notably, treatment group was identified as a significant factor, with patients in Group A demonstrating improved overall survival compared to Group B (HR = 3.66, 95% CI: 1.20-11.14, *p* = 0.036), after adjusting for confounding variables.

**Table 3:** Univariate and multivariate analysis of OS in breast cancer patients.

Variable	Univariate analysis		Multivariate analysis	
	HR (95%CI)	P-value	HR (95%CI)	P-value
Age (months)	1.02 (0.99, 1.05)	0.174	–	–
Menopausal status				
Premenopausal	reference	reference	–	–
Postmenopausal	1.65 (0.74, 3.66)	0.217	–	–
ECOG				
0	reference	reference	reference	reference
1	1.99 (0.77, 5.14)	0.157	2.07 (0.71, 5.99)	0.152
2	5.23 (1.47, 18.67)	0.011*	4.99 (1.17, 21.29)	0.024*
Metastatic pattern				
Metachronous disease	reference	reference	–	–
Synchronous disease	0.62 (0.73, 6.60)	0.514	–	–
PR				
Negative	reference	reference	reference	reference
Positive	0.42 (0.20, 0.88)	0.022*	0.49 (0.16, 1.55)	0.613
HER2				
zero	reference	reference	–	–
low	0.95 (0.44, 2.09)	0.908	–	–
Ki67				
≤ 30%	reference	reference	–	–
>30%	0.74 (0.32, 1.67)	0.463	–	–
Metastasis lesions				
1	reference	reference	reference	reference
2	2.15 (0.72, 6.46)	0.172	1.00 (0.23, 4.35)	0.614
≥ 3	3.10 (1.21, 7.93)	0.018*	2.41 (0.79, 7.57)	0.163
Bone metastasis				
No	reference	reference	–	–
Yes	0.94 (0.45, 1.93)	0.86	–	–
Visceral metastasis				
No	reference	reference	reference	reference
Yes	5.02 (1.92, 13.13)	0.001**	4.85 (1.52, 15.42)	0.013*
Radiotherapy				
No	reference	reference	reference	reference
Yes	3.14 (1.31, 7.49)	0.010*	2.58 (0.52, 12.84)	0.023*
Chemotherapy				
No	reference	reference	–	–
Yes	2.38 (0.56, 10.23)	0.241	–	–
Surgery on primary tumor				
No	reference	reference	–	–
Yes	0.41(0.15,1.13)	0.083	–	–
Sensitivity to endocrine therapy				
Primary resistance	reference	reference	–	–
Secondary resistance	0.80 (0.31, 2.04)	0.644	–	–
Sensitive	0.60 (0.19, 1.86)	0.372	–	–
Combined endocrine therapy				
Letrozole	reference	reference	–	–
Exemestane	0.85 (0.30, 2.35)	0.748	–	–
Anastrozole	0.28 (0.07, 1.08)	0.065	–	–
Fulvestrant	0.81 (0.32, 2.08)	0.667	–	–
Group				
Group B	reference	reference	reference	reference
Group A	0.85 (0.40, 1.80)	0.669	3.66 (1.20, 11.14)	0.036*

ECOG, Eastern Cooperative Oncology Group performance status; ER, estrogen receptor; PR, progesterone receptor; HER2, human epidermal growth factor receptor 2.

### 3.3 Subsequent treatments after using palbociclib

**Figure 3:**
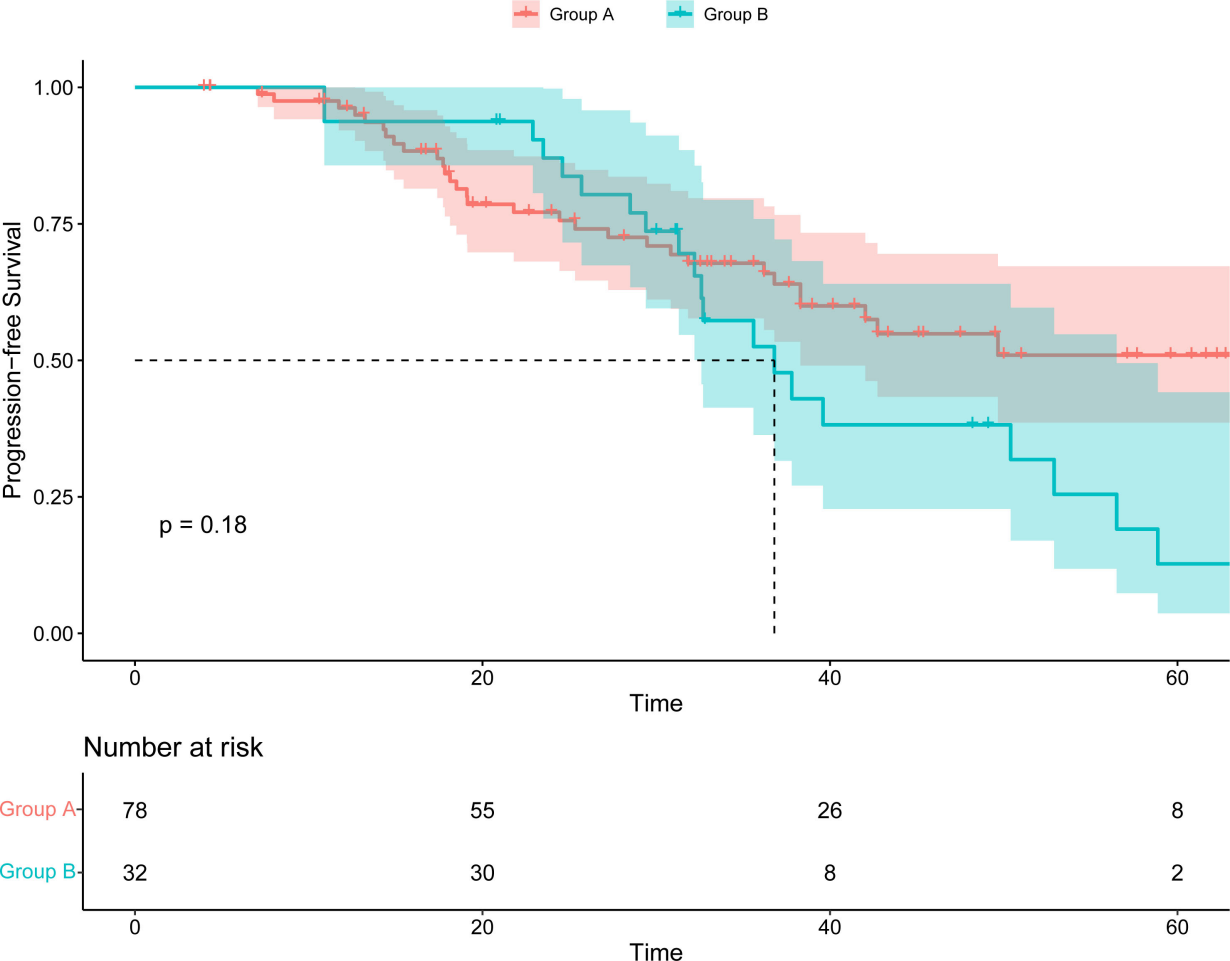
Kaplan–Meier survival curve of the cumulative PFS (the time from the date of advanced first-line treatment to the progress after advanced second-line treatment).

Following the application of palbociclib, progression was observed in 38 patients in group A, and 23 patients elected to undergo chemotherapy, yielding a median PFS of 7.83 months (95% CI, 6.00 to 11.80). Concurrently, a median PFS of 6.70 months (95% CI, 5.80 to NA) was documented for five patients who selected an alternative CDK4/6 inhibitor. Additionally, ten patients received endocrine-targeted therapy, registering a median PFS of 10.20 months (95% CI 5.07 to NA). The comparative analysis of PFS among the three groups revealed no significant disparity (*p*=0.3) (**Supplementary Figure 1**). Furthermore, an assessment of the cumulative PFS (the time from the date of advanced first-line treatment to the progress after advanced second-line treatment) between the two groups was conducted. Group A exhibited a median cumulative PFS of 30.15 months, whereas Group B demonstrated a median cumulative PFS of 32.4 months. The variance in cumulative PFS between Group A and Group B was not statistically significant (**Figure 3**).

### 3.4 Subgroup analysis

**Figure 4:**
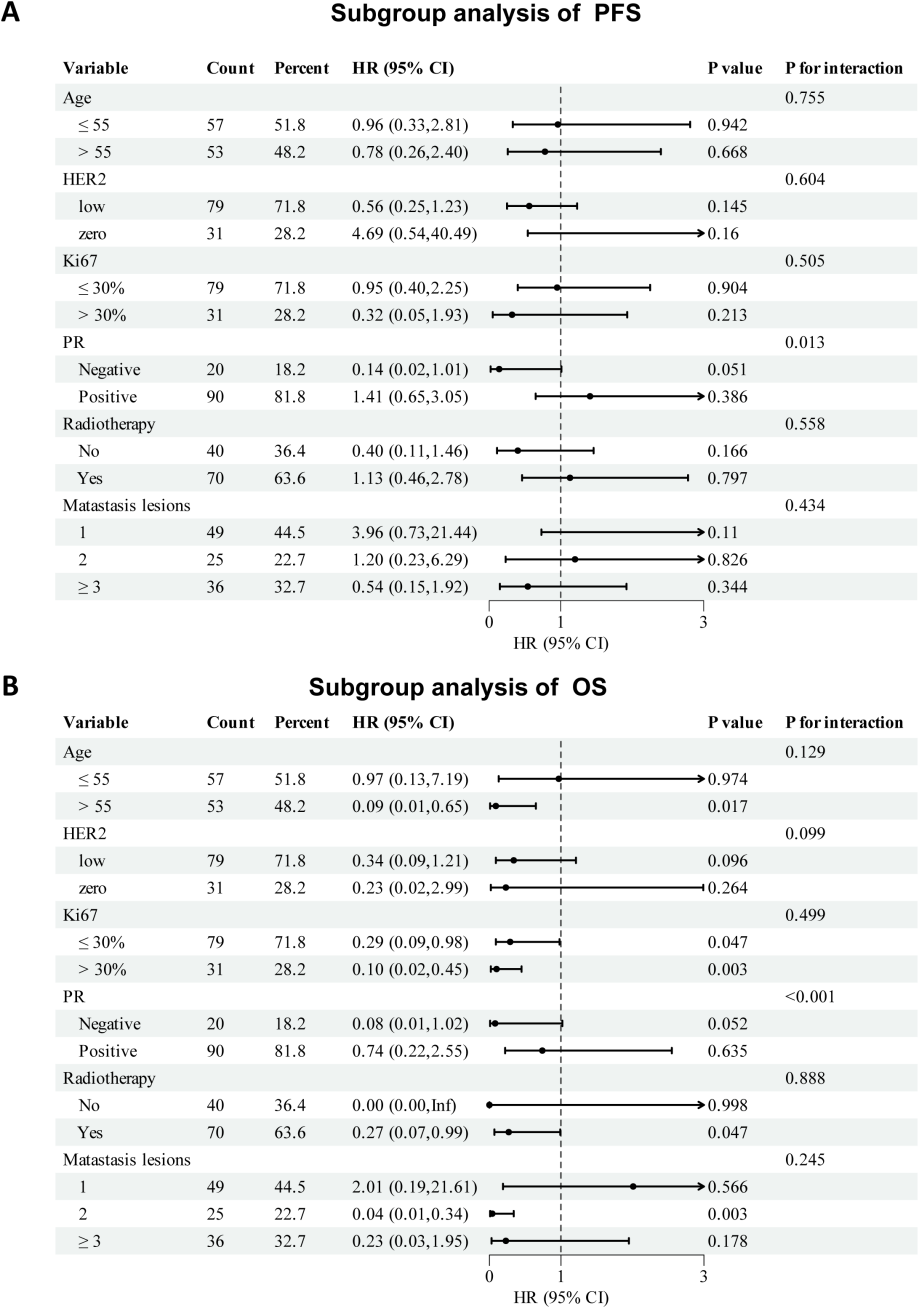
Subgroup analysis of **(A)** progression-free survival, **(B)** overall survival.

Subgroup analyses of PFS and OS are shown in **Figure 4**. The therapeutic effect of palbociclib-based treatment was generally consistent across most subgroups. For PFS, notable benefits were observed in PR-negative patients (HR: 0.14, 95% CI: 0.02–1.01, p = 0.051; P for interaction = 0.013) and those with ≥3 metastatic lesions (HR: 0.54, 95% CI: 0.15–1.92). Other subgroups, including age, HER2 status, Ki67 index, radiotherapy history, and bone metastasis, showed no significant interaction. In OS analysis, all subgroup HRs favored the palbociclib group, except in patients aged ≤55 years. Significant OS improvement was observed in patients aged >55 years (HR: 0.09, 95% CI: 0.01–0.65), those with high Ki67 (HR: 0.10, 95% CI: 0.02–0.45), and those who received radiotherapy (HR: 0.27, 95% CI: 0.07–0.99). PR status also showed a significant interaction effect (P < 0.001). Due to a small sample size and limited number of events, the treatment effect on HR in the subgroup of patients should be interpreted with caution.

### 3.5 Drug toxicity

Treatment-related adverse events were common but generally manageable in both groups (**Table 4**). The most frequent hematologic toxicities were neutropenia and leukopenia, observed in 94.3% and 91.4% of patients, respectively. Anemia was reported in 51.4% of patients overall, with no notable difference between the two groups. Non-hematologic adverse events were mostly grade 1 or 2 and included rash (37.1%), fatigue (17.1%), elevated liver enzymes (ALT 14.3%, AST 8.6%), oral mucositis (11.4%), and constipation (11.4%). Other less common toxicities such as nausea, diarrhea, alopecia, and hyperbilirubinemia were observed in fewer than 10% of patients. No treatment-related deaths or unexpected safety signals were reported. The incidence of adverse events was largely similar between the two groups.

**Table 4:** Incidence of treatment-related adverse events.

Toxicity	Total (N=110)	Group A (n=78)	Group B (n=32)
Neutropenia	104 (94.3%)	74 (94.9%)	30 (93.8%)
Leukopenia	101 (91.4%)	72 (92.3%)	29 (90.6%)
Anemia	57 (51.4%)	40 (51.3%)	17 (53.1%)
Rash	41 (37.1%)	29 (37.2%)	12 (37.5%)
Fatigue	19 (17.1%)	13 (16.7%)	6 (18.8%)
ALT elevation	16 (14.3%)	11 (14.1%)	5 (15.6%)
Oral mucositis	13 (11.4%)	9 (11.5%)	4 (12.5%)
Constipation	13 (11.4%)	9 (11.5%)	4 (12.5%)
AST elevation	9 (8.6%)	6 (7.7%)	3 (9.4%)
Thrombocytopenia	9 (8.6%)	6 (7.7%)	3 (9.4%)
Alopecia	9 (8.6%)	6 (7.7%)	3 (9.4%)
Nausea	6 (5.7%)	4 (5.1%)	2 (6.3%)
Diarrhea	6 (5.7%)	4 (5.1%)	2 (6.3%)
Hyperbilirubinemia	6 (5.7%)	4 (5.1%)	2 (6.3%)
Hand-foot syndrome	3 (2.9%)	2 (2.6%)	1 (3.1%)
Hyperglycemia	3 (2.9%)	2 (2.6%)	1 (3.1%)

ALT, Alanine Aminotransferase; AST, Aspartate Aminotransferase.

## 4 Discussion

HR-positive, HER2-negative breast cancer represents the most prevalent molecular subtype, accounting for over half of invasive breast cancers. ET has long been the cornerstone of treatment, significantly reducing recurrence and improving survival. However, both intrinsic and acquired resistance to ET remain major challenges, with up to 40% of early-stage patients ultimately progressing to metastatic disease (24). Despite advancements in hormonal agents such as tamoxifen, aromatase inhibitors, and fulvestrant, overall survival in MBC remains limited (25–31).

Consequently, improving survival outcomes for patients with HR-positive, HER2-negative MBC has become a critical therapeutic goal. Recent efforts have focused on targeted therapies, particularly CDK4/6 inhibitors and agents targeting the PI3K/AKT pathway (13, 32). Current guidelines support the use of CDK4/6 inhibitors in combination with ET as the standard first-line approach for patients without visceral crisis (33–40). Nonetheless, in select patients exhibiting rapid disease progression despite the absence of visceral crisis, chemotherapy remains a clinically relevant consideration. The optimal timing and patient selection for initiating CDK4/6 inhibitors with ET in such scenarios continues to be an area of investigation.

Hence, empirical research in real-world settings holds considerable importance. Our investigation assessed the determinants influencing therapeutic approaches and their efficacies. The baseline between the two treatment groups reflects the real-world clinical decision-making process. Patients in Group B, who received a short course of chemotherapy in addition to palbociclib and endocrine therapy, had a higher burden of disease at baseline, including more frequent visceral involvement, a greater number of metastatic lesions, and worse ECOG performance status. These clinical features likely influenced the physician’s choice to intensify treatment with chemotherapy, suggesting a more aggressive disease phenotype. Despite these unfavorable prognostic features, no significant difference in endocrine sensitivity or HER2 expression profile was observed between groups, indicating that molecular characteristics alone did not dictate treatment selection.

In this study, ECOG performance status and visceral metastasis were identified as independent predictors of OS. Although ECOG performance status 2 was found to be an independent predictor of poor survival in our multivariate analysis, this result should be interpreted with caution due to the small number of patients (n = 7) in this subgroup. The limited sample size may reduce the robustness of this finding. Further validation in larger, prospective cohorts is warranted to confirm the prognostic value of ECOG 2 in this population. Visceral involvement also predicted poor survival, consistent with its role as a marker of aggressive disease. Although PR expression (41), number of metastatic lesions, and radiotherapy history were significant in univariate analysis, they did not remain independent in the multivariate model, suggesting potential confounding. Notably, treatment group remained a significant factor, with better outcomes observed in patients receiving endocrine therapy plus palbociclib alone. This may reflect differences in baseline characteristics or treatment tolerance, though potential selection bias should be acknowledged. While the analysis of HER2-low versus HER2-zero expression did not reveal statistically significant differences in survival outcomes. This molecular distinction is gaining clinical relevance, particularly in light of novel HER2-targeted agents for HER2-low breast cancer. A recent multicenter retrospective study also found the same results (42). The absence of significance in our study may be due to limited sample size or real-world heterogeneity, but future studies with larger cohorts may better clarify its prognostic and therapeutic value. These findings underscore the importance of performance status, disease burden, and evolving molecular classifications in informing clinical decision-making for HR-positive, and HER2-negative MBC.

The safety profile observed in our cohort was consistent with the known toxicities associated with palbociclib and endocrine therapy. Neutropenia and leukopenia remained the most prominent hematologic adverse events, in line with previous clinical trials and real-world studies (43). Importantly, the majority of these cytopenias were manageable with dose modifications and supportive care, and did not lead to treatment discontinuation. The incidence of adverse events was largely similar between the two groups, indicating that the addition of short-course chemotherapy did not result in a substantially higher toxicity burden.

These results align with previous research, further supporting the evolving treatment paradigm of CDK4/6 inhibitors combined with endocrine therapy in HR-positive, HER2-negative MBC. The ABIGAIL Phase II trial presented by ESMO 2024 suggested that abemaciclib plus ET may outperform paclitaxel in short-term response rates, offering a chemotherapy-sparing alternative in selected patients. The MONALEESA-3 (36) and Korean ribociclib trials (44) reinforced the benefit of combining CDK4/6 inhibitors with ET across diverse patient subsets, including postmenopausal and premenopausal populations. Additionally, a comprehensive meta-analysis (45) encompassing 140 studies and 50,029 patients ascertained that in HR-positive, HER2-negative postmenopausal MBC patients. PFS did not significantly improve whether chemotherapy was administered with or without targeted therapy, in contrast to CDK4/6 inhibitors conjoined with ET. The Right Choice study (46) posited that initial treatment with a CDK4/6 inhibitor (ribociclib) plus ET conferred a notable PFS advantage, comparable response rates. And this study enhanced tolerability over combination chemotherapy in patients with clinically aggressive HR-positive, HER2-negative MBC. Consequently, the integration of CDK4/6 inhibitors with ET is advocated as the preferred initial treatment modality for HR-positive, HER2-negative MBC patients, including those with visceral metastases.

Our real-world research served as a foundation for therapy choices for patients with advanced, first-line HER2-negative, HR-positive cancer. However, several limitations are acknowledged: Primarily, palbociclib was the exclusive CDK4/6 inhibitor evaluated, thus not representative of the entire class of CDK4/6 inhibitors. Secondly, the study’s dataset was limited by baseline imbalances between the two treatment groups. Although multivariate Cox regression analysis was performed to adjust for these differences, the retrospective and observational design of the study means that residual confounding cannot be completely excluded. Future prospective studies with better-matched baseline characteristics and larger sample sizes—particularly within key subgroups—are needed to confirm and strengthen these findings. Lastly, the pandemic’s influence engendered irregular follow-up assessments for certain patients, potentially postponing the recognition of disease progression and, by extension, affecting the PFS measurements.

## 5 Conclusion

Derived from the outcomes of the above study and previous data, it is evident that the commencement of therapy with palbociclib plus ET was preferable to palbociclib plus ET after effective chemotherapy. This is consistent with previous study results and will provide real-world data support for the initial endocrine treatment of this group of patients.

## Data Availability

The datasets presented in this article are not readily available because The data that support the findings of this study concern patients’ privacy. Requests to access the datasets should be directed to Li Xiangjun, qddxlxj1004@163.com.
