# Supplementary figures and images for: A real-world study of palbociclib plus endocrine therapy with or without a short course chemotherapy in the first-line treatment of HR-positive HER2-negative metastatic breast cancer
Source: Front Oncol. 2025 Jul 9;15:1512496. doi: 10.3389/fonc.2025.1512496 (PMC12283718; doi:10.3389/fonc.2025.1512496)

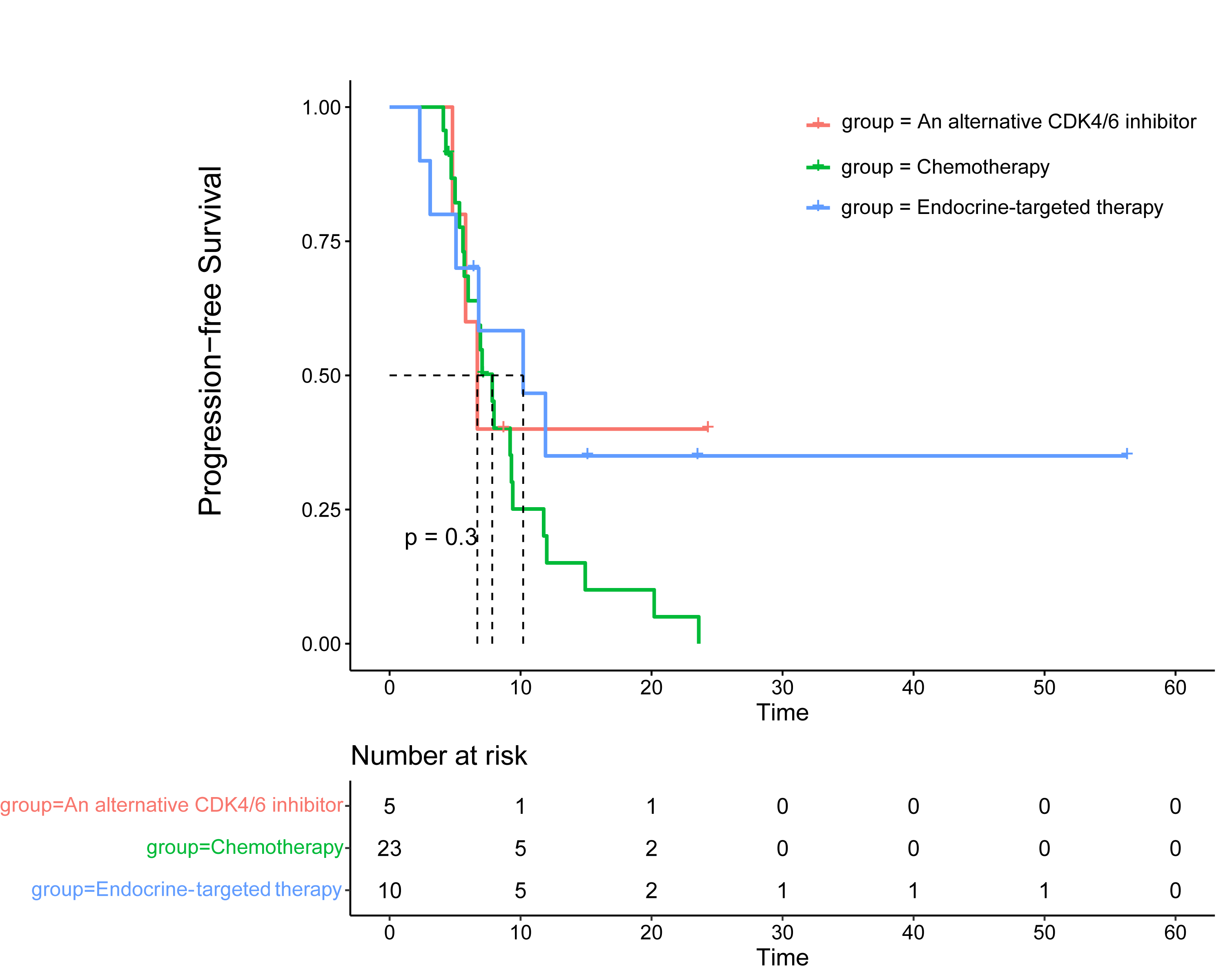

Supplement: Supplementary file 1 [file Image1.tif]
